# Inferring biological functions and associated transcriptional regulators using gene set expression coherence analysis

**DOI:** 10.1186/1471-2105-8-453

**Published:** 2007-11-17

**Authors:** Tae-Min Kim, Yeun-Jun Chung, Mun-Gan Rhyu, Myeong Ho Jung

**Affiliations:** 1 Division of Metabolic Disease, Center for Biomedical Science, National Institute of Health, Nokbun-dong 5, Eunpyung-gu, Seoul, Republic of Korea; 2 Department of Microbiology, College of Medicine, The Catholic University of Korea, Seoul, Republic of Korea; 3 Integrated Research Center for Genome Polymorphism, College of Medicine, The Catholic University of Korea, Seoul, Republic of Korea; 4 School of Oriental Medicine, Pusan National University, Busan, Republic of Korea

## Abstract

**Background:**

Gene clustering has been widely used to group genes with similar expression pattern in microarray data analysis. Subsequent enrichment analysis using predefined gene sets can provide clues on which functional themes or regulatory sequence motifs are associated with individual gene clusters. In spite of the potential utility, gene clustering and enrichment analysis have been used in separate platforms, thus, the development of integrative algorithm linking both methods is highly challenging.

**Results:**

In this study, we propose an algorithm for discovery of molecular functions and elucidation of transcriptional logics using two kinds of gene information, functional and regulatory motif gene sets. The algorithm, termed gene set expression coherence analysis first selects functional gene sets with significantly high expression coherences. Those candidate gene sets are further processed into a number of functionally related themes or functional clusters according to the expression similarities. Each functional cluster is then, investigated for the enrichment of transcriptional regulatory motifs using modified gene set enrichment analysis and regulatory motif gene sets. The method was tested for two publicly available expression profiles representing murine myogenesis and erythropoiesis. For respective profiles, our algorithm identified myocyte- and erythrocyte-related molecular functions, along with the putative transcriptional regulators for the corresponding molecular functions.

**Conclusion:**

As an integrative and comprehensive method for the analysis of large-scaled gene expression profiles, our method is able to generate a set of testable hypotheses: the transcriptional regulator X regulates function Y under cellular condition Z. GSECA algorithm is implemented into freely available software package.

## Background

Advanced high-throughput microarray technologies have facilitated the investigation of gene expression in a genome-wide manner [1,2]. Because of the complex nature and large volume of data, whole-genome expression profiles often require appropriate and comprehensive analytic methods. Gene clustering according to the expression similarity has been popularly used in this perspective, often as the first step of analysis [3]. In addition, functional enrichment analysis or pathway analysis was proposed to explain the global gene expression changes in the context of available knowledge, such as functional annotation of genes [4]. A classical enrichment analysis uses functionally annotated gene sets *a priori *defined from external gene databases (functional gene sets) and cross-references them with over- or under-expressed genes [5,6]. The use of enrichment analysis can be extended for different kinds of biological insights. For example, co-expressed genes grouped by clustering algorithm are likely to be regulated by common transcriptional control [3]. By using another type of gene set classified by the presence or absence of known transcription factor binding sites (TFBS) in promoter regions (regulatory motif gene sets), it can identify overrepresented TFBS with the corresponding putative transcriptional regulators [7,8].

In spite of promising utility, the conventional enrichment analysis dealing with individual gene clusters has several limitations. First, the size of gene clusters or gene sets is often so small that the statistical evaluation is prone to ascertainment bias, *i.e*. the significance of enrichment for small gene sets are frequently over- or underestimated. The advanced type of enrichment analysis, gene set enrichment analysis (GSEA) overcame this limitation by dealing with the entire genes represented by array as ranked gene list ordered by phenotypic correlation [9,10]. However, GSEA is suited for the comparison of two dichotomous phenotypic classes such as tumor versus normal, limiting its general use with gene clustering. Second, the accumulating biological knowledge on genes substantially increased the number of available gene sets to be used in enrichment analysis. Although recently proposed enrichment analysis tools can generate rich descriptions with the help of extended gene sets [11-13], they often produce unmanageably large lists for candidate gene sets to be considered especially when dealing with a large number of clusters. Rigorous statistical evaluation with the correction for multiple tests adjustment might be helpful to some extent, however, the development of integrative method is highly challenging to make the results more comprehensive.

In this study, we propose a method of gene set expression coherence analysis (GSECA) to provide a more advanced solution than the mere combining of gene clustering and enrichment analysis. The algorithm first selects functional gene sets with significantly high expression coherence as biologically relevant candidates for the corresponding expression profiles. Then, gene set clustering further reduces them into a number of functionally related gene sets, or functional clusters. On each functional cluster, putative transcriptional regulators are further identified using modified GSEA algorithm and regulatory motif gene sets. To demonstrate the applicability of our algorithm, we used two publicly available time-series gene expression profiles of the murine myogenesis and erythropoiesis. For respective profiles, our algorithm identified a number of functional themes and putative transcriptional regulators largely consistent with previous reports. As comprehensive and integrative method, GSECA algorithm has extended applicability for the analysis of multiple microarray expression datasets.

## Results and Discussion

### The overview of GSECA

The primary goal of GSECA algorithm is the discovery of molecular functions along with the elucidation of transcriptional regulatory logics for the interpretation of microarray datasets. For this purpose, two kinds of gene information – functional annotations in public gene database and the presence of regulatory motif sequences, or TFBS in the promoter regions – are used in terms of functional and regulatory motif gene sets, respectively. GSECA is composed of three major steps: selection of gene sets with significantly high expression coherence, clustering of functional gene sets into functional clusters and the identification of regulatory motifs associated with individual functional clusters.

**Figure 1:**
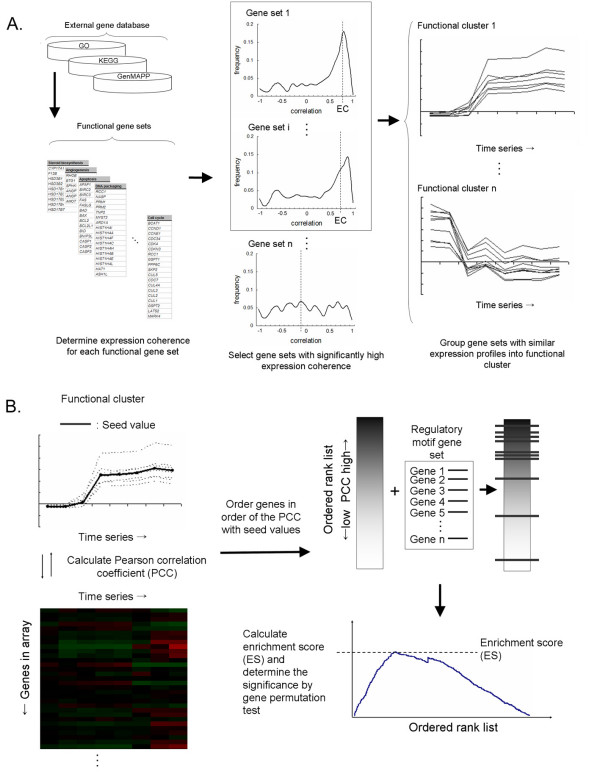
**Schematic representation of GSECA algorithm**. A. The individual steps of functional clustering are demonstrated. For each functional gene set prepared from public gene database (left), all pairs of gene members are calculated for Pearson correlation coefficient (PCC). The distribution of individual PCC is shown as histogram indicating how closely gene members are correlated with each other (middle). The mean of PCC values is calculated as expression coherence (EC) and the significance level is determined using gene permutation tests. Functional gene sets with significantly high expression coherences are then selected and grouped into respective functional clusters with similar expression patterns (right). B. Mean expression values of all genes belonging to the functional cluster are calculated as seed values of the corresponding cluster (left). The entire genes in the array are calculated for their similarity or Pearson correlation coefficient (PCC) with the seed values and ordered according to the similarity. The ordered gene list is then matched with regulatory motif gene sets and the extent of enrichment (enrichment score or ES) is determined by GSEA method (right).

First, GSECA determines whether gene members belonging to a predefined functional gene set are correlated with each other across the gene expression profiles (Fig. 1A). To do this, GSECA calculates the mean of Pearson correlation coefficient (PCC) for all pairs of gene members. The average PCC measure is used as the expression coherence of the corresponding gene set and it indicates how closely gene members are correlated with each other. The significance level for expression coherence level is then determined by gene permutation tests with adjustment for multiple tests. The functional gene sets with significantly high expression coherence are selected and the identified functional annotations are assumed to indicate the putative functionalities for which genes have coordinated expression changes across the different time points or experimental conditions.

Some of the candidate functional gene sets showed similar expression changes, making it possible to group them into a number of clusters. Thus, GSECA further categorizes those gene sets into several clusters using conventional clustering methods such as hierarchical or K-means clustering algorithm. The mean expression values of gene sets are used for the clustering and the gene sets with similar expression patterns are assigned into respective functional clusters. The functional annotations of gene sets assigned into a single functional cluster are also likely to represent similar molecular functions or pathways. Thus, this clustering reduces a collection of functional gene sets into more comprehensive set of functional clusters, and we refer collectively to these procedures as "functional clustering".

For each functional cluster, GSECA further identifies putative transcriptional regulators responsible for the expression patterns of the individual functional clusters. For this, GSECA exploits modified GSEA algorithm with regulatory motif gene sets predefined according to the presence of known TFBS in their promoter regions (Fig. 1B). To apply the GSEA algorithm, seed expression values of a functional cluster are first calculated for each time point by averaging the expression values of all genes belonging to the functional cluster. The entire genes in the array are then ordered according to the expression similarity or PCC with the seed values to make a ranked gene list. In the list, genes whose expression changes are similar to the seed values become top-positioned. The gene members of a regulatory motif set are then matched to the ordered rank list and measured for the enrichment using GSEA algorithm [9]. The significance level of enrichment is determined by gene permutation tests. The use of PCC as gene ordering metric is one of distinguishing features in GSECA algorithm and also extends the applicability of the conventional GSEA algorithm for the analysis of time-series expression profiles.

### Application of GSECA to murine myogenesis and erythropoiesis expression profiles

Cellular differentiation represent a series of intricate and complex cellular events the majority of which are under the control of transcriptional regulation. Therefore, time-series gene expression profiles derived from an *in vitro *cell differentiation model are good candidates for the application of GSECA algorithm. For test sets, we selected two kinds of publicly available time-series expression profiles representing the differentiation of murine myocytes [14] and erythrocytes [15]. First, we selected 1,206 functional gene sets including 5 – 100 genes and calculated expression coherence for each functional gene set. Significance level of expression coherence was determined by gene permutation tests and adjusted for multiple tests. As a result, 31 and 18 functional gene sets with significantly high expression coherence (*P *< 0.05, Bonferroni corrected) were identified in myogenesis and erythropoiesis expression profiles, respectively. We further used hierarchical clustering to classify functional gene sets with similar expression patterns into individual functional clusters.

**Figure 2:**
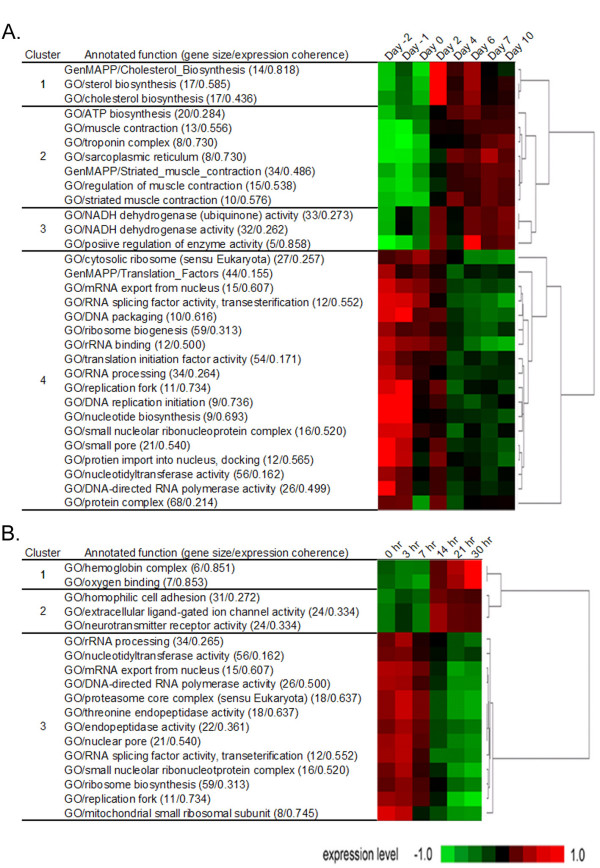
**Functional clustering of murine myogenesis- and erythropoiesis-related functional gene sets**. A. Thirty-one functional gene sets with significantly high expression coherences in myogenesis-related expression profile, are categorized into 4 functional clusters. For individual functional gene sets, gene numbers and expression coherence of the corresponding gene sets are also demonstrated in parentheses. Hierarchical clustering was used to measure the distances between functional gene sets and those with similar expression patterns were grouped into individual functional clusters. The expression level of a functional gene set is the mean expression value of the genes belonging to the gene set and schematically illustrated in heat map with gene set dendrogram. B. Three functional clusters composed of 18 functional gene sets are similarly demonstrated for erythropoiesis-related expression profile.

The 31 myogenesis-related functional gene sets were assigned into 4 functional clusters. Among the clusters, 7 functional gene sets with muscle-related functional annotations showed active transcriptional up-regulation after the induction of myogenesis and they were assigned into functional cluster 2 (Fig. 2A). It is not surprising that muscle-related functional gene sets are captured as one of key clusters in myogenesis-related expression profiles. However, it proves that our algorithm is able to identify the primary functional theme of interests, which would be beneficial in searching for perturbation-related molecular functions. In addition, the expression patterns observed for functional cluster 1 and 3 were distinguished from those of functional cluster 2. Two kinds of functional annotations – cholesterol biosynthesis and enzymatic activities of NADH dehydrogenase – were identified for functional cluster 1 and 3, respectively. These functions are likely to propose the additional functionalities associated with myogenesis in terms of cellular components and energy metabolism.

It has been known that genes with general housekeeping functions such as ribosomal genes, tend to be strongly correlated in expression profiles without direct evidence for their phenotypic association [16,17]. This is also the case of myogenesis dataset and the majority of functional gene sets identified with significantly high expression coherence (58%, 18/31 gene sets) were indicative of general housekeeping functions such as nucleotide or protein metabolism. Our study shows that the genes with housekeeping functions have correlated expression patterns not only at the individual gene level but also at the gene set level. Thus, it is reasonable to collectively treat them as a single functional cluster representing general housekeeping function (functional cluster 4).

Among the 18 erythropoiesis-related functional gene sets (Fig. 2B), two gene sets with characteristic functions of red blood cells – oxygen binding and hemoglobin complex – were assigned into functional cluster 1. Higher expression coherence of the two gene sets suggests that the genes with red blood cell function have coordinated and marked transcriptional up-regulation across the process of erythropoiesis. In addition, three gene sets with heterogeneous molecular functions such as cell adhesion and neurotransmitter receptor activity, were assigned into another functional cluster 2. Although speculative, those functions might present the potential functionalities with collaborative roles in erythropoiesis or hematopoiesis. Likewise the case of myogenesis, 13 functional gene sets representing the housekeeping functions showed similar expression changes throughout the erythropoiesis and they were collectively grouped into functional cluster 3.

### Identification of putative transcriptional regulators with modified GSEA algorithm and regulatory motif gene sets

The next step of GSECA is to identify the regulatory motif gene sets associated with individual functional clusters, which might propose the putative transcriptional regulators of the corresponding functional cluster. In case of myogenesis-related functional clusters, the cluster 2 with key annotations of muscle functions, showed significant enrichment (*P *< 0.05, Bonferroni corrected) for regulatory motif gene sets representing six transcription factors of Arnt, SREBP-1, Sp-1, MyoD, E2A, and USF (Table 1). Among them, MyoD is a well known transcription factor whose role in myogenic differentiation has been previously established [18,19]. This finding is consistent with that functional cluster 2 is composed of a set of muscle-related functional gene sets. The other transcription factors with significance enrichment might propose putative transcription regulators with regulatory roles in myogenesis, *i.e*. Sp-1 have some evidences on their co-activator role with MyoD factors in muscle-specific gene expressions [20,21]. For erythropoiesis-related functional cluster 1, three regulatory motifs such as SREBP-1, USF and GATA-1 were significantly enriched. In this case, GATA-1 was notable because the expression profile was derived from experiments in which GATA-1-null cell lines (G1E) are restored for their GATA-1 activity [15], supporting the biological relevance of regulatory motifs identified by GSECA algorithm.

**Table 1:** List of regulatory motif gene sets significantly enriched in individual functional clusters

Dataset	Functional cluster	Transcription factor^a^
Myogenesis	1	Sp-1
	2	Arnt, SREBP-1, Sp-1, MyoD, E2A, USF
	3	Sp-1, USF, LBP-1, Myc
	4	NRF-1, E2F, ATF/CREB, ETF, NF-Y, GABP, Elk-1, ZF5

Erythropoiesis	1	SREBP-1, USF, GATA-1
	2	AP1
	3	NF-Y, NRF-1, ATF/CREB, E2F, Arnt, Tel-2, Egr-3, Myc, ETF, Sp-1, GABP, YY1, HIF-1, Elk-1, ZF5

^a^Significantly enriched (*P *< 0.05, Bonferonni corrected) regulatory motif gene sets are shown for the corresponding transcription factors. When more than one regulatory motif sets corresponding to a single transcription factor were identified, the most significant one was listed taking the redundancy of regulatory motif gene sets into consideration. The listing order of transcription factors is according to the significance level of enrichment in individual functional clusters.

In addition, the functional cluster 4 of myogenesis profile representing the housekeeping functions showed enrichment for multiple ubiquitous transcription factors such as NRF-1, E2F, CREB, NF-Y, and ZF5. This is also the case of functional cluster 3 of erythropoiesis-related expression profile. The enrichment of multiple transcription factors might indicate the ubiquitous nature of the corresponding factors associated with general housekeeping functions [22,23]. However, the heterogeneity of functional gene sets might have also caused the enrichment of multiple regulatory motifs because the gene sets with housekeeping functions are manually assigned into a single cluster.

### Synergistic motif pairs in murine myogenesis and erythropoiesis

Transcription regulation among higher eukaryotes is likely to be mediated by multiple transcription factors in combinatorial modes rather than by a single agent [24]. In this perspective, the transcription factors that showed significant enrichment with functional clusters are good candidates for such potential synergism. Thus, we further investigated the synergistic relationship between regulatory motifs identified in previous step, *i.e*. 6 regulatory motifs enriched in functional 2 (myogenesis) and 3 motifs in functional cluster 1 (erythropoiesis). Motif synergy was called when genes belonging to both regulatory motif gene sets have significantly high expression coherence (*see *Methods). In case of myogenesis, three motif pairs (Arnt – SREBP-1, Sp-1 – MyoD and Sp-1 – E2A) involving five transcription factors were observed to have potential synergistic relationships (Table 2). Considering the evidences on the synergistic action between Sp-1 and MyoD [20,21], these motif pair sets might have possible combinatorial roles for the cellular process of myogenesis. In case of erythropoiesis, SREBP-1 and USF were observed to have putative synergistic relationship. Such relationship provides good candidate for the further transcription analysis associated with the erythropoiesis, given the previous evidences for their relationship in the transcriptional control of genes involved in lipid metabolism [25,26]. It must be noted that *in silico *analysis-yielded putative candidates cannot be assigned directly to functionality; however, it suggests the putative synergism between transcription factors and provides a testable set of hypotheses: transcription factors X_1 _and X_2 _might play a synergistic role for function Y under cellular condition Z.

**Table 2:** List of putative synergistic motif pairs

Dataset	Motif 1 (gene size/EC)^a^	Motif 2 (gene size/EC)	Gene size^b^	EC	Significance^c^
Myogenesis	Arnt (694/0.0020)	SREBP-1 (839/0.0024)	382	0.0086	0.02
	Sp-1 (3178/0.0006)	MyoD (696/0.0082)	318	0.0201	< 0.01
	Sp-1 (3178/0.0006)	E2A (906/0.0050)	444	0.0043	< 0.01

Erythropoiesis	SREBP-1 (1126/0.0171)	USF (372/0.0053)	839	0.0348	< 0.01

^a^Two regulatory motif gene sets are demonstrated as motif 1 and motif 2 with the gene numbers and expression coherence (EC) of the corresponding gene set. For motifs pairs, genes occurred both in two regulatory motif gene sets are separately measured for gene number^b ^and expression coherence. The significance level^c ^determined by permutation tests is also demonstrated. Only the motif pairs with significant expression coherence (*P *< 0.05) are shown in the list.

### Comparison of GSECA results with conventional enrichment analysis

**Figure 3:**
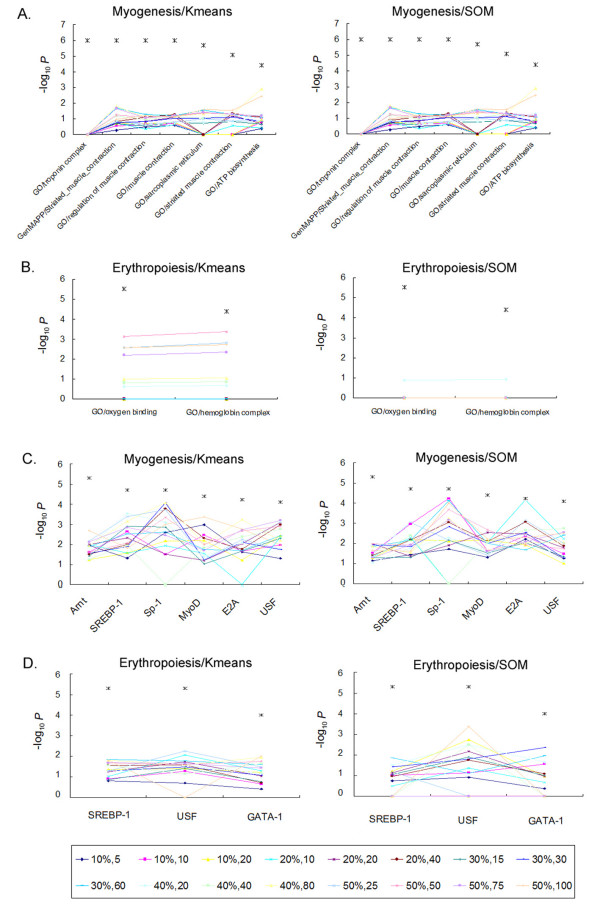
**Statistical comparison of GSECA results with conventional strategy**. A. Gene clustering and enrichment analysis was performed for 7 functional gene sets corresponding to the functional cluster 2 of myogenesis dataset. K-means and SOM clustering was performed with 16 different settings for the gene numbers to be clustered (5 – 50%) and cluster numbers (5 – 100). The significance levels (*Y*-axis) are illustrated with the color lines corresponding to 16 settings (shown in the bow below). For comparison, the unadjusted significance level or normal *P *value of GSECA algorithm are demonstrated as asterisk. B. The significance level for 2 functional gene sets of erythropoiesis are similarly calculated and compared with those of GSECA results. C and D. The comparison results of 6 and 3 regulatory motif gene sets with significance enrichment in the functional cluster 2 of myogenesis (C) and cluster 1 of erythropoiesis (D) are similarly demonstrated.

To demonstrate the advantages of GSECA, we performed the conventional strategy in which gene clustering and enrichment analysis are separately performed. For two test expression datasets, gene clustering was first performed using two commonly used gene partitioning algorithm of K-means and self-organizing maps (SOM). Clustering was done with diverse setting for the gene numbers to be clustered (5 – 50% of total genes) as well as the number of clusters (5 – 100 clusters), which fits in the conventionally used setting. For test, we selected 7 and 2 functional gene sets representing the characteristic functions of myogenesis- and erythropoiesis-expression profiles, respectively. The comparison results are demonstrated in Figure 3.

In case of myogenesis-related 7 functional gene sets, enrichment analyses combined with K-means or SOM clustering both yielded low level of significance which did not reach the threshold level of GSECA (unadjusted *P *< 4 × 10^-5^). This is also the case of erythropoiesis-related two functional gene sets. One plausible explanation for this low level of significance is the small size of functional gene sets in that functional gene sets containing less than 10 genes (*i.e*., troponin complex and sarcoplasmic reticulum) showed the lowest level of significance. In case of 2 gene sets in erythropoiesis, they both have less than 10 genes and showed variable level of significance across the different settings or used clustering methods. This is consistent with our initial assumption that conventional enrichment analysis dealing with small gene cluster or gene sets might be prone to over- or under-estimation of the significance.

The significance for enrichment of regulatory motif gene sets were also improved in GSECA analysis as shown for 6 and 3 gene sets for myogenesis and erythropoiesis expression profiles, respectively. The significance of enrichment for biologically relevant regulatory motifs such as MyoD and GATA-1 is two to three folds higher in GSECA results. The improved statistical power in detecting the regulatory motifs of interest might be due to the modified GSEA algorithm used in our method [9,10,27]. The adoption of modified GSEA algorithm is likely to provide the robustness and sensitivity of the advanced GSEA algorithm as possible explanation for improved statistical power over the conventional methods.

### Considerations on GSECA methodology

The initial assumption of GSECA is that functional gene sets with significantly high expression coherence suggest putative functionality. It must be noted that annotated functions of gene sets with higher expression coherences do not always correspond directly with the actual biological functions [17]. Nonetheless, many physiological cellular responses require the simultaneous participation of gene products and genes with central roles are likely to have similar regulatory control and expression patterns [28-30]. Comparative analysis also showed that co-expression patterns of many functionally-related genes are conserved across diverse species [31]. Thus, gene sets with significantly high expression coherence might, if not all, represent the key molecular functions of the corresponding expression profiles.

Our algorithm also concerns how the functionality represented by functional clusters can be linked to regulatory motifs to elucidate the putative transcriptional regulators. Cares must be taken in that genes collected from the functional gene sets assigned to a functional cluster might not fully represent the putative transcriptional targets considering that the current functional gene annotation is not complete. To compensate for this, GSECA implements a modified GSEA algorithm to exploit the entire gene expression profiles in terms of correlation with seed values of functional clusters. Similarity-based gene ordering along with the enrichment algorithm is likely to ensure the robustness and sensitivity of GSEA algorithm as demonstrated by the comparison with conventional strategy.

The use of GSEA algorithm also facilitates the adoption of the extended application for GSEA algorithm recently proposed to increase the statistical power or for improved biological insights. For example, by using absolute correlation as ordering parameter, GSEA can detects unique functional categories whose gene members have both extreme transcriptional up- and down-regulation [32]. If such strategy can be applied in GSECA algorithm, it can detect putative regulatory motifs with dual roles of transcriptional enhancers and inhibitors in the cellular contexts. However, one distinguishing feature of GSECA, the use of distance metric such as PCC also limits the use of GSECA algorithm only for time- or condition-series expression profiles as compared with conventional GSEA which is oriented for the comparison of two phenotypic classes.

We also provide an additional method to identify putative synergistic motif pairs among multiple transcription factors. The method has been previously introduced and used to identify the synergistic combination between transcription factors in yeast [33] and human [34]. However, due to the large number of regulatory motif gene sets in pairwise combination and permutation tests to be considered, the method is often not feasible for general application. Thus, it would be beneficial to select a subset of putative regulatory motifs to reduce the computational work load and GSECA can provide such plausible candidates for the in-depth analyses of combinatorial actions between transcription factors. Expression coherence-based identification of motif synergy would provide clues on the complex structure of regulatory modules and substrates for further experimental validation [35]. However, recent studies on the elucidation of transcription regulatory networks use more sophisticated network assumptions and detailed parameters on the motif sequences and their relationships [36,37]. Moreover, *in silico *analysis-based results and significances must be interpreted with care because they do not always represent the actual functionality or causality.

In addition, there have been efforts to incorporate the biological knowledge into the gene clustering to maximize the statistical efficiency and reliability of the analysis results. For example, functional gene annotations can be directly incorporated in the distance metric [38], or used to guide the clustering procedures [39,40]. However, most methods in this perspective use the functional GO categories as additional information for fine-tuning of distance metrics to optimize the clustering, or to evaluate the results of conventional clustering algorithms [41]. By contrast, GSECA algorithm directly calculates the expression coherences of predefined gene sets then, categorizes into a number of functional clusters by gene set clustering. Gene set-based clustering used in GSECA provides an additional advantage over the conventional strategy in which gene clusters are individually measured for enrichment with functional or regulatory motif gene sets, *i.e*. improved statistical power and comprehensive interpretation of the results.

## Conclusion

In this study, we address an integrative method for the interpretation of multiple expression profiles in terms of two kinds of gene information; function gene annotation and sequence information of TFBS in the regulatory regions. It measures two kinds of parameters, expression coherence and the extent of enrichment in similarity-based ranked gene list to identify the putative functionality and transcription regulators, respectively. Our method successfully identified the key molecular functions and putative transcriptional regulators for two test expression profiles, which were largely consistent with the literature-based knowledge. With improved statistical power over the conventional strategy, our algorithm has extended applicability for rich descriptions of high-throughput microarray expression data.

## Methods

### Test expression profiles

Examples of microarray datasets were downloaded from public expression databases, Gene Expression Omnibus or NCBI GEO [42]. We used two expression datasets representing time-scaled gene expression changes for the differentiation of murine myocytes (accession no. GDS586 in GEO database) [14] and erythrocytes (GDS568) [15]. Both datasets were prepared using the same expression microarray platform of Affymetrix MG-U74Av2 with similar hybridization protocols [43]. The global expression profiles were median-centered and normalized to set the sum of the squares of probe intensities to be 1.0. We used NetAffx Gene Ontology Mining Tools [44] to intersect the used probes into Entrez gene annotation. Through the study, we used Entrez gene annotation as the common link for functional and regulatory motif gene sets.

### Preparation of functional and regulatory motif gene sets

We used NetAffx software for the functional categorization of genes to prepare the function gene sets. The gene grouping was based on functional annotations in public gene databases, GO (Gene Ontology), KEGG (Kyoto Encyclopedia of Genes and Genomes) and GenMAPP (Gene Map Annotator and Pathway Profiler) [45-47]. A regulatory motif gene set or a TFBS-annotated gene set is defined as a set of genes containing the sequence motif for corresponding TFBS in their regulatory regions at least once. To prepare regulatory motif gene sets from a publicly available TFBS annotation database [48], the precomputed fingerprint files were downloaded from Expander package [7,49]. This database includes the information of putative *cis*-regulatory sequences predicted based on experimentally validated binding sequence information for known transcription factors. In total, 432 TFBS-annotated regulatory motif gene sets were prepared as previously described [8] and used for enrichment analyses.

### Functional clustering using expression coherence of functional gene sets

For each functional gene set, GSECA first determined the extent of how gene members in a gene set might correlate with each other. As distance measure, GSECA calculated the Pearson correlation coefficient (PCC) for all possible pairs of genes, omitting self-comparisons. The mean value of PCC was used as the "expression coherence" of the functional gene set. For biological relevance, we only used gene sets containing 5 – 100 highly variable genes, because too few genes might lead to selection bias, and the functional annotation of large gene sets is commonly indicative of non-informative general function. To determine the significance level for expression coherence, we used gene permutation tests. For each gene set with *n *number of genes, expression coherence was calculated for *n *randomly selected genes, and the fraction of random sets that acquired higher expression coherence in 10^6 ^tests was determined as a *P *value. The nominal *P *values were adjusted for the multiple testing with Bonferroni correction accounting for the number of functional gene sets. For functional gene sets with significantly high expression coherence, mean expression values of the gene members belonging to the gene set were calculated for each time point. Then, agglomerative hierarchical clustering was used to classify the functional gene sets with similar expression patterns by using the PCC as distance measure. We defined such clustered functional gene sets as individual "functional clusters".

### Identification of transcriptional regulators for functional clusters

For each functional cluster, we collected the gene members included in the functional gene sets of the corresponding functional cluster. Mean expression values across different time points were calculated as "seed" values of representative expression changes for the functional cluster. Then, using regulatory motif gene sets, we identified putative transcriptional regulators responsible for the seed expression values of individual functional clusters. The overall procedure is similar to that described for the conventional GSEA algorithm [9], while the most distinguishing feature of GSECA is that it uses PCC as the gene ordering parameter, rather than signal-to-noise ratio (SNR). First, the entire genes in the array were calculated individually for the similarity of expression to the seed values of each functional cluster in terms of the PCC. Then, the genes were ordered according to the PCC and the genes with higher PCC or those being more similar to seed values are top-ranked in the ordered gene list. Regulatory motif gene sets were matched to such gene lists, calculating enrichment score (ES) using Kolmogorov-Smirnov statistics [9]. The significance level for ES was calculated using 5 × 10^5 ^gene permutation tests and adjusted for multiple testing accounting for the number of regulatory motif gene sets. In conventional GSEA algorithm, phenotypic permutation is preferred in that gene to gene correlation is preserved [9,10]. However, phenotypic permutation is often not feasible for common time-series expression datasets due to the small number of samples. To demonstrate that gene permutation tests can obtain the biologically relevant findings, we used gene permutation in adopting modified GSEA algorithm. However, it must be noted that gene permutation often overestimates the significance levels [10].

### Identification of synergistic motif pairs using expression coherence

Pairs of putative transcriptional regulators acting in combinatorial mode were investigated using previously described method [33,34]. For a candidate pair of two regulatory motifs, expression coherence was calculated for all pairs of gene members that occurred both in two regulatory motif gene sets. The significance level for the expression coherence was measured by gene permutation tests. For expression coherence of *n *number of genes that occurred both in two regulatory motif gene sets, two sets of the same number of genes were randomly selected from two regulatory motif gene sets and expression coherence is calculated. The nominal *P *value was calculated as the fraction of random sets that acquired higher expression coherence in 5,000 permutation tests.

### Comparison of significance level with conventional strategy

For conventional strategy in which gene clustering and enrichment analysis are separately performed, we used two commonly used partitioning cluster algorithms, K-means and SOM (self-organizing maps). We tried 15 different settings for variable number of genes to be clustered (5 % – 50 % of ~10,000 total genes) and numbers of clusters (5 – 100 clusters). For each setting, the individual clusters were measured for the enrichment with the same functional and regulatory motif gene sets used in GSECA. The significance of enrichment was measured using hypergeometric distribution:

p=1−∑i=0k−1(Mi)(N−Mn−i)(Ni),
 MathType@MTEF@5@5@+=feaafiart1ev1aaatCvAUfKttLearuWrP9MDH5MBPbIqV92AaeXatLxBI9gBaebbnrfifHhDYfgasaacPC6xNi=xI8qiVKYPFjYdHaVhbbf9v8qqaqFr0xc9vqFj0dXdbba91qpepeI8k8fiI+fsY=rqGqVepae9pg0db9vqaiVgFr0xfr=xfr=xc9adbaqaaeGacaGaaiaabeqaaeqabiWaaaGcbaGaemiCaaNaeyypa0JaeGymaeJaeyOeI0YaaabCaeaajuaGdaWcaaqaamaabmaabaWaaSaaaeaacqWGnbqtaeaacqWGPbqAaaaacaGLOaGaayzkaaWaaeWaaeaadaWcaaqaaiabd6eaojabgkHiTiabd2eanbqaaiabd6gaUjabgkHiTiabdMgaPbaaaiaawIcacaGLPaaaaeaadaqadaqaamaalaaabaGaemOta4eabaGaemyAaKgaaaGaayjkaiaawMcaaaaaaSqaaiabdMgaPjabg2da9iabicdaWaqaaiabdUgaRjabgkHiTiabigdaXaqdcqGHris5aOGaeiilaWcaaa@4B6D@

*N *and *M *is the total number of genes in array and cluster gene numbers, *n *is the size of corresponding gene set and *k *is the number of genes both occurred in gene set and cluster. For each setting, the most significant enrichment across the clusters was selected and assigned to the individual functional and regulatory motif gene sets.

### Implementation of GSECA algorithm

The overall procedures of GSECA are implemented into freely available software. The test files with two expression profiles along with functional and regulatory motif gene sets (human and mouse) are also available with the software package. The software package and technical manual can be downloaded in our website.

## Availability and requirements

Project name: GSECA

Project home page: 

Operating system: Microsoft Windows

Programming language: VB.NET

Other requirements: .NET Framework 2.0 or greater

License: None

Any restrictions to use by non-academics: None

## Authors' contributions

TMK and MHJ conceptualized and designed the algorithm. TMK and YJJ analyzed the data. MGR and MHJ wrote the manuscript. All authors read and approved the final manuscript.
